# Better Phonological Short-Term Memory Is Linked to Improved Cortical Memory Representations for Word Forms and Better Word Learning

**DOI:** 10.3389/fnhum.2020.00209

**Published:** 2020-06-05

**Authors:** Sari Ylinen, Anni Nora, Elisabet Service

**Affiliations:** ^1^CICERO Learning, Faculty of Educational Sciences, University of Helsinki, Helsinki, Finland; ^2^Cognitive Brain Research Unit, Department of Psychology and Logopedics, Faculty of Medicine, University of Helsinki, Helsinki, Finland; ^3^BioMag Laboratory, Helsinki University Central Hospital, Helsinki, Finland; ^4^Department on Neuroscience and Biomedical Engineering, Aalto University, Espoo, Finland; ^5^ARiEAL Research Centre, Department of Linguistics and Languages, McMaster University, Hamilton, ON, Canada

**Keywords:** magnetoencephalography, phonological short-term memory, language learning, paired-associate word learning, phonological mapping negativity

## Abstract

Language learning relies on both short-term and long-term memory. Phonological short-term memory (pSTM) is thought to play an important role in the learning of novel word forms. However, language learners may differ in their ability to maintain word representations in pSTM during interfering auditory input. We used magnetoencephalography (MEG) to investigate how pSTM capacity in better and poorer pSTM groups is linked to language learning and the maintenance of pseudowords in pSTM. In particular, MEG was recorded while participants maintained pseudowords in pSTM by covert speech rehearsal, and while these brain representations were probed by presenting auditory pseudowords with first or third syllables matching or mismatching the rehearsed item. A control condition included identical stimuli but no rehearsal. Differences in response strength between matching and mismatching syllables were interpreted as the phonological mapping negativity (PMN). While PMN for the first syllable was found in both groups, it was observed for the third syllable only in the group with better pSTM. This suggests that individuals with better pSTM maintained representations of trisyllabic pseudowords more accurately during interference than individuals with poorer pSTM. Importantly, the group with better pSTM learned words faster in a paired-associate word learning task, linking the PMN findings to language learning.

## Introduction

Phonological short-term or working memory (pSTM) has been suggested to play a critical role in language learning, contributing to the establishment of long-term memory traces (for reviews, see Gathercole and Baddeley, 1993; Baddeley et al., 1998; Service, 2013). A number of studies have shown a link between pSTM and the learning of first-language and foreign vocabulary (e.g., Service, 1992; Service and Kohonen, 1995; Atkins and Baddeley, 1998; Gathercole et al., 1999) and syntax (French, 2006; French and O’Brien, 2008). In the working memory framework by Baddeley (1986) see also Baddeley and Hitch (1974), all speech has obligatory access to a phonological short-term store, and its contents are refreshed by a rehearsal component that prevents the decay of memoranda. According to Baddeley et al. (1998), this phonological loop is critical for word learning because it is used to maintain unfamiliar sound patterns in memory while more permanent memory representations are being constructed. An alternative view has questioned the direction of causality, suggesting instead that vocabulary size may determine pSTM capacity (e.g., Melby-Lervåg et al., 2012). Longitudinal studies of second-language learning that have followed the accumulation of vocabulary from the start have lent support to the original view by Baddeley, Gathercole and colleagues (Service and Kohonen, 1995; French, 2006). Recently, brain stimulation studies have linked the storage component of pSTM, housed in the left supramarginal gyrus, to the ability to support the maintenance of verbal order (Papagno et al., 2017; Savill et al., 2019). The ability to represent the order of phonemes in a novel word form, and the order of words in phrases, has been suggested as the mechanism relating pSTM to learning of both the phonological structure of novel word forms and grammatical phrases (Gupta and Tisdale, 2009).

At the level of neuroanatomy, the phonological loop was first suggested to rely on Broca’s area and the left supramarginal gyrus (Paulesu et al., 1993; for more recent work, see Papagno et al., 2017; Savill et al., 2019). Later neuroimaging studies on pSTM point to a network involving also posterior temporal or temporo-parietal areas [posterior superior temporal gyrus (STG), posterior superior temporal sulcus (STS), posterior planum temporale (PT), or Sylvian-parietal-temporal areas (Spt)] areas (Buchsbaum et al., 2001; Hickok et al., 2003; Buchsbaum and D’Esposito, 2008; McGettigan et al., 2011; Richardson et al., 2011; Herman et al., 2013), also supported by lesion studies (Baldo et al., 2012). Extending the phonological loop model, rehearsal has been suggested to be a process of circulating information between phonological input and output buffers, involving temporo-parietal cortex and left inferior frontal cortex, respectively (Jacquemot and Scott, 2006; Herman et al., 2013). A framework for STM maintenance and language repetition by Majerus (2013) proposes that speech is encoded and phonological representations maintained in fronto-temporal language networks (dorsal and ventral speech processing streams; Scott and Johnsrude, 2003; Hickok and Poeppel, 2007) and attentional focalization is coordinated from a fronto-parietal network.

Linking the neural implementation of auditory working memory or pSTM with word learning, both short-term and longer-term changes related to establishing memory representations for novel words have been shown in auditory cortices (Davis and Gaskell, 2009; Davis et al., 2009). Thus, when novel words are maintained in pSTM, cortical responses in temporal areas reflect the quality of the phonological memory representations, with contributions from frontal motor representations (Nora et al., 2015). In addition to neocortical areas, medial temporal areas have been shown to be important for initial encoding and maintenance during word learning, and a recent study by Kumar et al. (2016) demonstrates the involvement of hippocampus as well as fronto-temporal connections in all stages of the working memory process, namely, encoding, maintenance, and retrieval.

Encoding of speech input has been suggested to be the primary determinant for efficient pSTM functioning (Barry et al., 2009, 2011) and long-term learning (Service et al., 2007). However, also the maintenance of phonological information has been shown to exert an influence on remembering and learning words (Davachi et al., 2001). pSTM contents are thought to be affected by decay or interference but can be maintained for a longer period by rehearsal or attentional refreshing (i.e., focusing attention on memoranda for their maintenance; Camos et al., 2009; Camos and Barrouillet, 2014; Lewandowsky and Oberauer, 2015). When considering the learning of spoken words during natural communication, a typical source of interference is the auditory input following the to-be-learned word. In this case, efficient maintenance may strengthen the pSTM representation and protect it from interference. This raises questions whether individual learners differ in their ability to maintain word forms in pSTM during interfering auditory input, whether this is reflected in the cortical activation during phonological processing, and whether word learning varies as a function of this phonological maintenance ability and its neural correlates. Some previous studies (e.g., Čeponienë et al., 1999; Barry et al., 2009) have compared brain responses in participants with poorer and better pSTM. However, these studies neither used tasks with active pSTM maintenance during interference, nor actual word-learning tasks in adults.

We used magnetoencephalography (MEG) to study the maintenance of novel word forms in pSTM by rehearsal during interfering auditory input. Firstly, by comparing brain responses between participants that have better or poorer pSTM, we aimed to determine whether pSTM maintenance during interference differs between these groups. Secondly, to clarify how the ability to maintain phonological representations is the brain influences language learning, we investigated whether these groups differ in their word-learning ability. The experimental paradigm used here has been described in Ylinen et al. (2015). In each trial, participants first heard a target pseudoword that they were instructed to rehearse covertly. This target pseudoword was followed by random distractors as well as probe stimuli that fully or partially matched with the rehearsed target. This condition was compared with a control condition in which pseudoword rehearsal had been replaced by silent counting of recurring visual symbols (i.e., there was no match between pSTM contents and auditory stimulation). We assumed that the rehearsal of word forms in working memory would re-activate or refresh the phonological representations of to-be-remembered target pseudowords and protect them from interference caused by auditory distractors. Probe stimuli were used to test the level of activation and accuracy of these representations in participants with better or poorer pSTM.

The rationale of using probe stimuli is based on the findings that covert speech used in rehearsal in pSTM generates forward prediction of the rehearsed item, projected from frontal cortex speech areas to auditory cortex in the form of efference copy signals. Efference copies are internal copies of efferent commands produced by the motor system (cf. Sperry, 1950; von Holst and Mittelstaedt, 1950). The forward prediction appears to regulate the activation of auditory cortex (Houde et al., 2002; for covert speech see Tian and Poeppel, 2010, 2012; Ylinen et al., 2015). Auditory input matching overt or covert speech has been found to suppress responses in auditory cortex, whereas mismatching input enhances the responses (Chang et al., 2013; Ylinen et al., 2015). Thus, when covert rehearsal is combined with matching or mismatching auditory probe stimuli, brain responses can be used to index pSTM maintenance.

We were particularly interested in detecting neural activity that previous studies have linked with a discrepancy from phonological expectations. In tasks involving listening to wordlike stimuli while phonological expectations are active, specifically the event-related potential or field (ERP/ERF) component named the phonological mapping negativity (PMN, formerly phonological mismatch negativity) has been observed (Connolly and Phillips, 1994; D’Arcy et al., 2004; Kujala et al., 2004). PMN is elicited at about 200–250 ms after an unexpected phoneme is encountered and it has been located to anterior temporal cortex (Kujala et al., 2004). Enhanced responses are seen when phonological expectations based on sentence or phonotactic context or covert speech are not met. Therefore, PMN can be used to index pSTM maintenance by covert rehearsal. The PMN has been associated with phonology because it is similarly elicited for words and pseudowords, ruling out dependence on lexical-semantic processing for its elicitation (Newman and Connolly, 2009).

The PMN is thought to reflect mapping of auditory input onto phonemes in speech recognition, yet the results for distinguishing children with language or literacy disorders from typically developing children based on the PMN have been mixed (Bonte and Blomert, 2004; Desroches et al., 2013; Malins et al., 2013). However, it is noteworthy that previous work has been limited to inspecting mismatches in the first syllable. As discussed by Connolly et al. (2001), the PMN response is likely not limited to onset processing. Here we report responses to both salient onsets and less salient third syllables in trisyllabic stimuli. We predicted that auditory pseudowords mismatching the rehearsed pseudoword would elicit a stronger magnetic PMN response compared with pseudowords matching it. Moreover, we thought that the PMN process might be sensitive to pSTM abilities. We, therefore, hypothesized that the brain responses of groups with better and poorer pSTM might differ from each other at the PMN latency as activated phonological representations are thought to be necessary for PMN elicitation. When hearing distractors, participants with better pSTM were expected to show more accurate maintenance of phonological items due to more persistent and resilient phonological representations. In contrast, the groups’ responses were not expected to differ from each other in the control condition with no resemblance between internal speech and the auditory stimuli. We further thought that the less salient third syllables may be more sensitive to group differences (cf. Service and Maury, 2003). Finally, based on behavioral studies (Service and Kohonen, 1995), we also hypothesized that the neural correlates of pSTM should be linked to indices of language learning, such that if there are differences in PMN between the groups, similar differences should be found also in a paired-associate novel word learning task.

## Materials and Methods

### Pre-test

#### Ethics Statement

All subjects signed a written informed consent form before participation in the experiment. The pre-test was approved by the Ethical Committee of the Faculty of Behavioural Sciences, University of Helsinki.

#### Participants

Fifty-one university students [28 females, 23 males; age 19–34 years, mean age 23.8 years (y), *SD:* 4.09] volunteered for the pre-test. The inclusion criteria were right-handedness, normal hearing, no early bilingualism, Finnish as the native language, no language or speech disorders including dyslexia, and no neurological or psychiatric disorders or drug addiction. Three females were excluded from analysis because of these criteria.

#### Procedure

The pre-test included three cognitive tasks, with order of presentation counterbalanced within the participant group: (1) pseudoword pair repetition, (2) pseudoword memory span, and (3) paired-associate novel word learning. The pseudoword repetition task (Čeponienë et al., 1999) included 20 pairs of 4- and 5-syllabic pseudowords with relatively complex, but for Finnish legal, phonological structure (e.g., /sohraelma/–/nahterkop:io/). Participants were instructed to listen to the pseudoword pairs, then say “toistan” (I repeat) and then repeat aloud the pseudoword pairs. The initial “toistan” was intended to wipe echoic memory content. The pseudoword span task (Numminen et al., 2002) included lists of spoken disyllabic CVCV pseudowords for immediate recall. Participants were first presented with 10 lists of three pseudowords, then 10 lists of four pseudowords, and finally 10 lists of five pseudowords. After hearing a list, the participants’ task was to repeat back the list in the order of presentation. Word learning was studied with a paired-associate learning task with eight familiar Finnish words each paired with a Finnish-sounding pseudoword (Čeponienë et al., 1999). Four items were disyllabic and four trisyllabic. After reading each of the eight pairs, participants saw one Finnish word at a time and were instructed to say aloud the corresponding pseudoword. The task had four trials during which the same eight word-pseudoword pairs were presented in random order. Out of these tasks, pseudoword pair repetition and pseudoword memory span were used to assign participants to two pSTM groups for the MEG experiment (better or poorer pSTM). The paired-associate word learning task was, in turn, used to compare language learning ability between these groups.

#### Statistical Analysis of Paired-Associate Word Learning

Paired-associate word learning scores were submitted to a mixed ANOVA including within-subjects factors Word length (short, long), Trial (1, 2, 3), and a between-subjects factor Group (better pSTM, poorer pSTM).

### MEG Experiment

#### Ethics Statement

All subjects signed a written informed consent form before participation in the experiment. The MEG experiment was approved by the Research Ethics Committee of Helsinki University Central Hospital. All experiments were carried out according to the Declaration of Helsinki.

#### Participants

Based on pSTM performance in the pre-test, a standard compound score was formed by transforming pseudoword repetition and pseudoword span raw scores (the number of items repeated correctly and the number of lists repeated correctly, respectively) into z-scores for each task and participant and then averaging the z-scores across the tasks for each participant. Thirteen participants with the lowest scores and 13 participants with the highest scores were invited to take part in the MEG recording. They formed a poorer and a better pSTM group, respectively. Participants were not informed of the group they belonged to. One participant in each group was unavailable for the MEG session, resulting in 12 participants in the poorer pSTM group (six females and six males; mean age 23.08 years, *SD*: 2.98) and 12 in the better pSTM group (seven females and five males; mean age 23.67 years, *SD:* 4.62). The MEG participants’ standard pSTM score ranged from −2.61 to −0.58 in the poorer pSTM group and from 0.48 to 1.64 in the better pSTM group. The average raw score was 8.33 for pseudoword repetition and 6.67 for pseudoword span in the poorer pSTM group and 17.33 for pseudoword repetition and 16.83 for pseudoword span in the better pSTM group.

#### Stimuli

**FIGURE 1:**
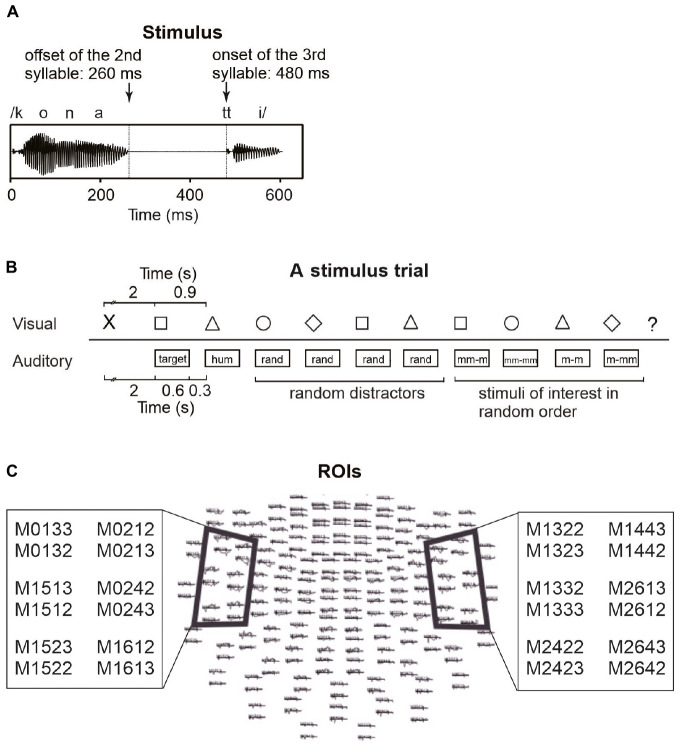
Stimulation and data analysis. **(A)** A waveform of an example stimulus and its timing. **(B)** A stimulus trial and its timing (rand: random distractor; mm-m: mismatching beginning, matching ending; mm-mm: mismatching beginning, mismatching ending; m-m: matching beginning, matching ending; m-mm: matching beginning, mismatching ending). **(C)** MEG channels above regions of interest (ROIs), used to crate areal mean signals (AMS). AMSs were calculated over six channels above the left and right temporal areas.

The auditory and visual stimuli in the MEG experiment were the same as those in Ylinen et al. (2015; see Figure 1A). The auditory stimulus material included 30 different pseudowords, each having two variants (60 stimuli in total). The pseudowords had a CVCVCCV structure (e.g., /pukot:o/, /tavek:o/, /konat:a/) with geminate stop consonants including a silent phase before the release burst in the third syllable. The pseudowords complied with the phonotactic structure of Finnish but were unfamiliar to the participants. The stimuli were produced at a normal speaking rate by a female native speaker of Finnish and digitally recorded with a Eurorack MX1604A Mixer and a Røde NT2-A microphone in an acoustically shielded room. The final experimental stimuli were chosen from several variants on the basis of judgments of three naïve native speakers of Finnish, who assessed the goodness of the stimuli with respect to their native language. The chosen pseudowords were further modified with Praat 5.0.40. (Boersma and Weenink, 2008) as follows: the intensity of the stimuli was scaled to 90% and the durations of the syllables within the stimuli were equalized preserving their typical ratio (the 1^st^ and 2^nd^ syllable excluding the final consonant 260 ms; the silent phase of the geminate stop 220 ms; the 3^rd^ syllable (excluding the silent phase of the stop) 120 ms; in total 600 ms, see Figure 1A). In addition to speech stimuli, the stimulus set included a humming sound that was created by filtering a pseudoword stimulus [pamup:a] with a 250 Hz low-pass filter and a harmonic tone of 75 ms duration and 500 Hz fundamental frequency (with harmonic partials of 1000 and 1500 Hz). Responses to these non-speech sounds were not analyzed.

The participants were also presented with visual stimuli on a screen in front of them (see Figure 1B) simultaneously with the auditory stimuli. The stimuli were geometric shapes (a square, a circle, a triangle, a diamond) displayed in black on a gray background. The stimulus presentation was commanded by a script written in Presentation 12.2 (Neurobehavioral Systems, Albany, NY, United States).

#### Procedure

The MEG experiment followed the procedure of Ylinen et al. (2015). There were two task conditions, rehearsal and control, in both of which participants heard stimulus pseudowords from loudspeakers and simultaneously saw visual symbols on the screen. In the rehearsal condition, participants were instructed to covertly rehearse the first auditory pseudoword of the trial each time an auditory stimulus was heard (and a simultaneous visual symbol was shown). To ensure that the participants rehearsed the heard items as instructed, they had to say the rehearsed psudoword aloud at the end of the trial when a question mark was shown on the screen. In the control condition, the participants’ task was to count the number of occurrences of the visual symbol that had been presented first in that trial. To ensure that the participants performed the task as instructed, they had to say aloud the number of counted symbols at the end of the trial when a question mark was shown on the screen. The two conditions were run in counterbalanced order within the two participant groups. The 100 trials of each condition were divided into five blocks, and 10 s breaks were inserted between the blocks. Participants were instructed to blink extensively during breaks to reduce blinking during the experimental trials. Each block started with the presentation of 20 repetitions of a harmonic tone, after which the task began. Participants were allowed to take a break between conditions. Instructions for the task in question were given immediately before each condition.

The tasks differed between the two conditions, but the stimulation was identical (with the exception that the order of the 30 pseudowords was randomized separately for the conditions). Each trial (see Figure 1B) consisted of a sequence as follows. First, a cross showed up on the screen as a signal to get ready to perform the task. After 2 s, the participants heard a pseudoword and saw the first geometric shape symbol. Depending on the condition, they were to remember and covertly rehearse the pseudoword, or to silently count occurrences of the symbol during the trial. Then a humming sound (a low-pass filtered pseudoword) was presented to set the rhythm for the covert rehearsal. A hum instead of another pseudoword was used to avoid immediately erasing the to-be-remembered pseudoword from phonological memory before the participants had got started with the rehearsal. After the hum, four random pseudowords that did not resemble the to-be-remembered word were presented as distractors. These were followed by four stimuli in random order. These included (1) the same pseudoword as the rehearsed word (but not identical recording), (2) a minimal-pair pseudoword with a different final vowel (e.g., for rehearsed pseudoword [pukot:a], minimal pair [pukot:o]), (3) a different pseudoword but with the same ending (e.g., for rehearsed pseudoword [pukot:a], a pseudoword with the same ending [konat:a]), and (4) a random pseudoword not resembling the rehearsed pseudoword (e.g., for rehearsed pseudoword [pukot:a], a random pseudoword [kilep:o]). All presentations of auditory pseudowords were accompanied by the simultaneous presentation of visual symbols, but the auditory and visual stimuli were not otherwise associated with each other. The same visual symbol was presented 2–4 times in random order during a trial. After 10 simultaneous presentations of auditory and visual stimuli, a question mark was shown on the screen, indicating that participants should say aloud, depending on the task, either the rehearsed pseudoword or the number of counted symbols in the trial. This was to make sure the participants were performing the tasks as instructed. Since the to-be-remembered pseudowords were followed by four auditory distractors and four other stimuli (see Figure 1B), it is unlikely that the participants could have remembered and said the pseudowords aloud, if they had not rehearsed them in pSTM (i.e., without active maintenance of the to-be-remembered pseudowords, the distractors would have erased them from phonological memory). A new trial started 2.5 s after the presentation of the question mark. Within each trial, the interstimulus interval was 300 ms.

#### MEG Recording and Analysis

ERFs were recorded with a 306-channel Vectorview MEG device (Elekta Neuromag, Elekta Oy, Helsinki, Finland) with 204 planar gradiometers and 102 magnetometers. Simultaneously, electroencephalography (EEG) was recorded from three scalp sites (Fz, Cz, and Pz, referenced to the left mastoid; EEG analysis is not reported here). The participants sat in a magnetically and acoustically shielded chamber with their head covered by the helmet of the MEG device. They were instructed to avoid blinking except during the breaks between the blocks and not to move their head (even during the breaks). Before the experiment, four head-position indicator coils were attached to each participant’s head and their location with respect to anatomical landmarks of the head (nasion and pre-aurical points) was determined by an Isotrak 3D digitizer (Polhemus, Colchester, VT, United States). The position of the head within the helmet of the MEG device was determined by feeding current to the coils and measuring their locations in the helmet. MEG and EEG signals were recorded with a 600 Hz sampling rate and filtered with a band pass of 0.1–200 Hz.

The data were off-line filtered with band pass of 0.5–30 Hz (slope 12 and 24 dB/octave, respectively) and artifacts exceeding 1200 fT/cm on gradiometers were rejected. Baseline was set to 100 ms windows preceding the onset of the analyzed syllables (−100 to 0 ms for the first syllable and 380–480 ms for the third syllable; cf. Barry et al., 2009). To determine response strength, areal mean signals (AMSs) were calculated from six gradiometer pairs above the temporal lobe of each hemisphere (see Figure 1C for channel locations) where the responses of interest were expected to be elicited (Ylinen et al., 2015). Time windows for analysis were selected based on the latencies of the highest grand-average AMS peaks in the time window of 150–300 ms from syllable onset (i.e., around PMN latency), determined separately for the four stimulus types (for time windows in each condition, see Table 1). 50 ms time windows were centered at the latencies of these peaks to calculate response strength for each experimental condition. These conditions included first-syllable match, first-syllable mismatch, third-syllable match, and third-syllable mismatch (i.e., rather than using mismatch-minus-match difference waves, response strengths were calculated from the AMS for each condition). The AMS for the first syllable matching with the target was the average across the responses to all the stimuli with the matching beginning (i.e., including pseudowords that were the same as the rehearsed target, e.g., target [pukot:a] vs. [pukot:a], and the stimuli that had the same beginning but mismatching ending, e.g., target [pukot:a] vs. [pukot:o]). The AMS for a mismatching first syllable was the average across the responses to all the stimuli with a mismatching beginning (i.e., including the pseudowords with mismatching beginning and ending, e.g., rehearsed target [pukot:a] vs. [kilep:o], and those with mismatching beginning but matching ending with respect to the target, e.g., target [pukot:a] vs. [konat:a]). The AMSs for the third syllable included responses to match (e.g., [pukot:a] vs. [pukot:a]) or mismatch (e.g., [pukot:a] vs. [pukot:o]) with respect to the third syllable of the rehearsed target (note that in both cases, the pseudoword beginnings matched the target until the third-syllable onset at 480 ms). We expected the items that had a mismatching first/third syllable with respect to the rehearsed target to elicit a stronger response at the PMN latency compared with the matching items. Moreover, we expected stronger PMN responses in the group with better pSTM capacity.

**TABLE 1:** Time windows used for quantifying areal mean signals (AMS).

Rehearsal condition								

	1^st^ syllable match		1^st^ syllable mismatch		3^rd^ syllable match		3^rd^ syllable mismatch	
	LH	RH	LH	RH	LH	RH	LH	RH
Better pSTM	147–197	190–240	188–238	192–242	648–698	646–696	666–716	656–706
Poorer pSTM	155–205	152–202	155–205	192–242	650–700	645–695	665–715	653–703

**Control condition**								

	**1^st^ syllable “match”**		**1^st^ syllable mismatch**		**3^rd^ syllable “match”**		**3^rd^ syllable mismatch**	

	LH	RH	LH	RH	LH	RH	LH	RH
Better pSTM	150–200	183–233	185–235	177–227	663–713	651–701	666–716	661–711
Poorer pSTM	153–203	200–250	148–198	192–242	699–749	653–703	655–705	665–715

*Note that in the control condition, there is no actual match to pSTM contents like in the rehearsal condition, yet the auditory stimuli are the same in the two conditions. LH, left hemisphere; RH, right hemisphere.*

To control for group differences in overall engagement in the rehearsal task, we also inspected the suppression effect of N1 caused by covert rehearsal in the third syllable. If participants were performing the rehearsal task, the covert rehearsal of items matching the auditory stimuli should induce suppressed N1 responses as compared to the control condition (Ylinen et al., 2015). If the N1 suppression effect was different between the groups, then the groups’ effort or engagement in rehearsal could have been different. AMSs were calculated from the same six gradiometer pairs as included for PMN, but only in the left hemisphere, where suppression effects at the syllable level were expected to occur (Ylinen et al., 2015). Time windows for analysis were selected based on the latencies of the highest grand-average AMS peaks at 100–140 ms from the 3^rd^ syllable onset. AMS peaks were determined separately for the different stimulus types, and 50 ms time windows were centered at the latencies of these peaks to calculate response strength for each experimental condition (time windows for N1 ranged from 575–625 to 586–636 ms, i.e., from 120 to 131 ms from the 3^rd^ syllable onset).

#### Statistical Analysis of AMS

For statistical analysis of AMS strength, we used mixed ANOVA with repeated factors Syllable (first, third), Task (rehearsal, control), Match [matching, mismatching with the rehearsed syllable (or equivalent stimulus in the control condition)], Hemisphere (left, right) and the between-subjects factor Group (better pSTM, poorer pSTM). In addition, we report step-down analyses with repeated factors Task (rehearsal, control), Match [matching, mismatching with the rehearsed syllable (or equivalent stimulus in the control condition)], Hemisphere (left, right) and the between-subjects factor Group (better pSTM, poorer pSTM). Consequent interactions were followed up with Bonferroni-corrected pairwise comparisons. To ensure that both groups were engaged by the rehearsal task in a similar manner, the suppression effect of N1 that is caused by covert rehearsal was compared between the groups by submitting N1 AMSs for the third-syllable match condition to an ANOVA including factors Task (rehearsal, control) and Group (better, poorer pSTM).

## Results

### Paired-Associate Word Learning

**FIGURE 2:**
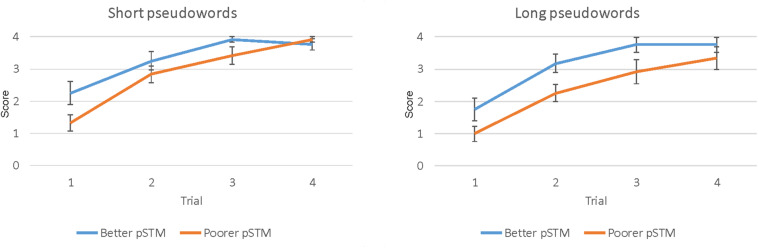
Paired-associate learning score (number of items correct ± SEM) of four short (left) and four long (right) pseudowords during four trials in participants with better or poorer pSTM.

The paired-associate word learning task had four trials. However, in the last trial both groups performed close to ceiling, indicating that this trial could not show group differences accurately (see Figure 2). Therefore, the results of trials 1–3 were used in the analysis. The mixed ANOVA showed the main effects of Word length [*F*(1,22) = 5.78, *p* = 0.025], with higher scores for shorter than longer words, Trial [*F*(2,22) = 71.93, *p* < 0.001], with higher scores on later than earlier trials, and Group [*F*(1,22) = 5.83, *p* = 0.025], with higher scores in the better than the poorer pSTM group (see Table 2 and Figure 2).

**TABLE 2:** Paired-associate word learning scores (±SD) averaged across trials 1–3 in particioants with better or poorer phonological short-term memory (pSTM).

	Short words	Long words
Better pSTM	3.13 (±0.78)	2.89 (±0.89)
Poorer pSTM	2.53 (±0.78)	2.06 (±0.81)

### Areal Mean Signals

**FIGURE 3:**
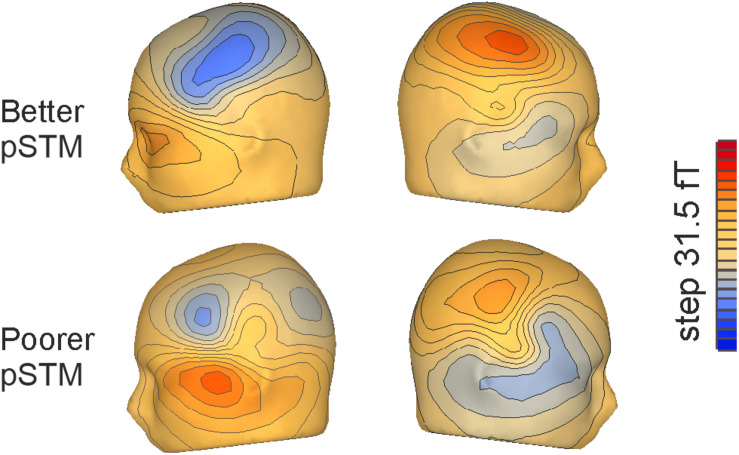
Flux maps for the third syllables of pseudowords mismatching the contents of covert rehearsal in the left and right hemisphere, respectively, in participants with better (top) and poorer (bottom) pSTM.

**FIGURE 4:**
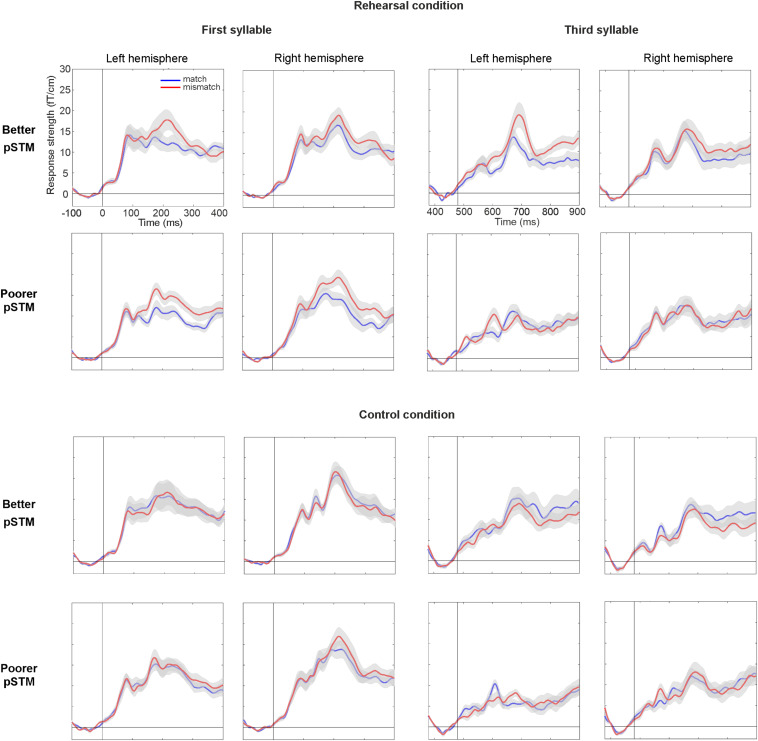
Areal mean signals (AMS, averaged across 6 gradiometer pairs located over temporal lobes) for the first syllable (left panel) and third syllable (right panel) in participants with better and poorer pSTM in rehearsal (top) and control (bottom) conditions. The standard error of the mean is shown in gray color around the AMS. Vertical line denotes syllable onset, whereas timescales are shown with respect to stimulus onset. In rehearsal condition, larger mismatch than match at around 200 and 700 ms for the first and third syllables, respectively, is interpreted to reflect PMN.

In line with previous PMN literature (Kujala et al., 2004), syllables mismatching the contents of covert rehearsal induced activity over anterior temporal cortex (see Figure 3). At the PMN latency, a five-way ANOVA with the AMS as dependent variable showed a significant four-way interaction of Group × Syllable × Task × Match [*F*(1,22) = 4.81, *p* < 0.039]. This was further explored by separate ANOVAs of responses to the first and the third syllable. In the first-syllable analysis, the main effect of Match was significant [*F*(1,22) = 23.51, *p* < 0.001] due to stronger responses to mismatching than matching stimuli. The main effects of Task [*F*(1,22) = 17.17, *p* < 0.001] and Hemisphere [*F*(1,22) = 9.35, *p* = 0.006] were also significant due to stronger responses for the control than rehearsal task and stronger responses over the right than left hemisphere, respectively. There was also a significant interaction of Task × Match [*F*(1,22) = 12.21, *p* = 0.002]. According to pairwise comparisons, the responses were significantly stronger to mismatch than match in both tasks (for control, *p* = 0.015; for rehearsal, *p* < 0.001). No interactions or effects involving Group were observed for the first syllable (see Figure 4).

In contrast, for the third syllable we found an interaction of Group × Task × Match [*F*(1,22) = 9.59, *p* = 0.005], which was due to significantly stronger responses to mismatching than matching stimuli in the better pSTM group in the rehearsal task (*p* < 0.024), but not in the control task (n.s.). Furthermore, a significant interaction of Group × Task × Hemisphere × Match [*F*(1,22) = 6.85, *p* = 0.016] showed that the rehearsal effect in the better pSTM group was driven by significantly stronger responses to mismatch than match in the left hemisphere (*p* = 0.013, see Figure 4). All other pairwise comparisons for this interaction were non-significant. In the group with poorer pSTM, Match comparisons were not significant in either task.

An ANOVA for the N1 component for the third syllable in the matching condition was run to establish that there were no group differences in the auditory effects of rehearsal as compared with the control task. Rehearsal would be expected to result in auditory cortex suppression effects in the matching condition. The analysis showed a significant main effect of Task [*F*(1,22) = 10.86, *p* = 0.003], but the effects of Group [*F*(1,22) = 0.057, n.s.] and Task × Group [*F*(1,22) = 0.143, n.s.] did not approach significance.

## Discussion

By presenting auditory probes matching or mismatching the word forms rehearsed in pSTM, the present study aimed to determine how pSTM ability affects the maintenance of word forms during interference and whether this ability and its neural correlates are linked to language learning. Firstly, two groups with different pSTM capacities were compared in paired-associate word learning of word-pseudoword pairs. Although both groups performed close to ceiling on the fourth trial, those with better pSTM had significantly higher learning scores during the first three trials. Thus, those with better pSTM learned the associations faster, with fewer repetitions. Secondly, comparison of AMSs over the temporal cortices did not suggest rehearsal-related differences between the pSTM groups in the processing of the first syllable of the pseudowords, yet only the better pSTM group showed a significant PMN response for a mismatch in the third syllable.

The maintenance of the phonological form of pseudowords in pSTM by covert rehearsal modulated responses peaking around the typical PMN latency, that is, about 200 ms from 1^st^ and 3^rd^ syllable onsets (about 200 and 680 ms from stimulus onset, respectively). In the rehearsal condition, the effect of covert rehearsal on the processing of the first syllable was reflected in a significantly stronger response to mismatching than matching stimuli in both groups. This is in line with earlier PMN findings (e.g., Connolly et al., 2001) as well as findings showing that matching covert speech suppresses auditory responses (Numminen and Curio, 1999; Kauramäki et al., 2010; Tian and Poeppel, 2012; Ylinen et al., 2015), whereas mismatching input elicits enhanced responses (Chang et al., 2013; Ylinen et al., 2015). However, only the better pSTM group showed an enhanced PMN response to a mismatch in the third syllable in the rehearsal condition. This result indicates that pSTM ability modulated the phonological processing accuracy of the endings of trisyllabic word forms. Different effects with respect to pSTM between the first and third syllables suggest that the role of pSTM in the processing of phonological sequences may differ between word beginnings and endings or between shorter and longer words, which is in line with previous results on phonological memory (Service and Maury, 2003). The pattern of results suggests that pSTM ability determines the accuracy of phonological representations for all phonemes of novel words during interfering input. Those with poorer pSTM may be able to represent accurately only short word forms or beginnings of longer word forms and be challenged to fully represent novel multi-syllabic word forms.

The group differences in representing trisyllabic word forms in pSTM as reflected by the PMN can be accounted for by differences in either pSTM maintenance (see Buchsbaum et al., 2005; Buchsbaum and D’Esposito, 2008) or encoding input into pSTM (Barry et al., 2011). Regarding possible group differences in pSTM maintenance, one may ask whether participants with poorer pSTM rehearsed the words less extensively despite the same instructions given to the two groups. Given that both N1 suppression and PMN enhancement may reflect the ongoing rehearsal process, such a difference does not seem likely because N1 for the probe that matched the rehearsed pseudoword appeared similarly diminished in both groups in the rehearsal condition. This suggests that the participants with poorer pSTM rehearsed the pseudowords covertly as requested and must have had at least some kind of active representations for the rehearsed pseudowords that could modulate N1. Why, then, would those with poorer pSTM be unable to maintain strong enough pSTM representations to elicit PMN in the third syllable despite rehearsal? One possible answer is that their pSTM capacity was overloaded by rehearsal of a trisyllabic novel form and processing of an incoming form with also three syllables. Together this task requires memory for six ordered syllables, if aligned third syllables are to elicit a mismatch response.

Another possibility is that since participants were requested to rehearse pseudowords covertly along with the rhythm of regularly presented stimuli, brain responses may have been modulated by the participants’ ability to synchronize their rehearsal with auditory input. A recent study by Assaneo et al. (2019) has suggested that individuals’ spontaneous ability to synchronize their speech to an isochronous train of auditorily presented syllables is linked to differences in white matter and brain-to-stimulus synchronization over frontal areas. However, rather than spontaneous synchronization, our task more closely resembles metronome-beat synchronized speech, where participants have been very accurate in keeping the external rhythm, with mean differences in actual and expected time between the productions being within 10 ms (Davidow et al., 2010). Although it is not clear to which extent PMN might be modified by synchronization abilities, previous studies have shown that synchronized rehearsal is not a prerequisite for PMN elicitation. The PMN is often elicited in a task where a word is first manipulated in one’s mind and then an auditory stimulus is presented afterward (see, e.g., Connolly et al., 2001). Thus, poor synchronization skills cannot fully account for the lack of third-syllable PMN response in the poorer pSTM group.

Besides pSTM maintenance by rehearsal, group differences in third-syllable PMN might also be influenced by differences in the encoding process of auditory stimuli to pSTM for rehearsal. Previous research by Barry et al. (2011) has suggested that encoding words into memory results in larger hemodynamic responses in individuals with better non-word repetition (pSTM ability). In another study, Barry et al. (2009) found that in an oddball paradigm, those with poorer non-word repetition had smaller late discriminative negativity (LDN) responses for pseudoword-internal third syllables of auditory stimuli, interpreting this to reflect less efficient encoding. In particular, they suggested that in poor non-word repeaters, syllable recognition is not rapid enough and, therefore, earlier syllables interfere with the processing of later syllables of longer words. Consistent pSTM effects in the processing of the third syllable across studies (i.e., the current study and Barry et al., 2009) support the view that memory capacity is linked to these effects via word length. We do not, however, find in our data any consistent differences between better and poorer pSTM groups in the pace of processing (see Table 1 and Figure 3). In addition, we found the group differences in responses to the final syllable. According to Barry et al. (2009), the processing of the final syllable should have recovered from a cumulative memory load effect in those with poorer pSTM, if their problem was a slower rate of the encoding process.

Nevertheless, it is still possible that the group differences were related to encoding, for example via the code used in pSTM maintenance. Although we have previously argued that the code of covert rehearsal in our task is most likely phonological (Ylinen et al., 2015), recent literature suggests that pSTM may use both acoustic storage and categorical representations (Joseph et al., 2015) and that items can be maintained by rehearsing phonologically or by using domain-general attentional refreshing (i.e., focusing attention on memoranda for their maintenance; Camos et al., 2009; Camos and Barrouillet, 2014; Lewandowsky and Oberauer, 2015). These studies suggest that phonological rehearsal is not a necessity for the maintenance of verbal material in pSTM. In a similar vein, our inner speech may vary with respect to the detail of its phonological formulation. Therefore, one possible account for our pattern of results is that participants in the poorer pSTM group used less phonological means of maintenance during pSTM tasks, for example by occasionally (or consistently) maintaining acoustic-phonetic representations via attentional refreshing. The code used in pSTM maintenance, in turn, could either be due to the efficiency of phonological encoding process or the efficiency of the maintenance process itself. Although this account is speculative in the sense that it was not part of our original hypothesis, it could explain the lack of third-syllable PMN in participants with poorer pSTM while at the same time they showed similar N1 effects for the third syllable as the better pSTM group. Further research is needed to clarify the effect of pSTM ability on the code used in pSTM.

Unexpectedly, ANOVA suggested stronger neural activation for mismatching than matching first syllables of the pseudowords also in the control condition. As illustrated by Figure 4, however, this difference is more subtle than in the rehearsal condition (particularly in the participants with better pSTM). Note that since the control condition included no rehearsal of pseudowords, the stimuli could not actually match memoranda maintained in pSTM. Therefore, there is no match and mismatch in the same sense as in the rehearsal task. However, in each trial there were two kinds of stimuli, the beginnings of which were phonologically identical (i.e., a stimulus that, in the context of rehearsal, would have had a matching beginning and ending or a matching beginning and mismatching ending; this design was necessary to study the third syllable), which might contribute to the effect. We can only speculate why responses to the two stimulus types differed in the control condition, but one possibility is that the presence of these two pseudowords with phonologically identical beginnings in the same trial interacted with attentional control. A previous study by Engell et al. (2016) has shown that sounds preceded by maskers with similar frequencies resulted in more reduced activation when participants attended to the auditory modality compared to when they attended to the visual modality. Perhaps, then, if our participants did not properly inhibit auditory stimuli that were irrelevant to the control task, repetition of phonologically identical pseudoword beginnings in close succession may have caught their attention, which in turn may have modulated their responses.

## Conclusion

The comparison of MEG responses in individuals with better or poorer pSTM suggested that pSTM capacity affected the ability to maintain pseudowords in phonological memory during interference, as reflected in PMN responses. Specifically, the maintenance of the third syllables but not the first syllables differentiated between poorer and better pSTM groups. It seems that tri-syllabic words challenge pSTM and, therefore, PMN responses to these longer words can reveal differences in pSTM capacity. We also found that those with better pSTM and stronger third-syllable responses learned words faster in a paired-associate word learning task, suggesting a link between pSTM maintenance (or encoding and maintenance) and language learning. This might be related to use of a phonological code in the maintenance of spoken word forms and their phoneme order in pSTM.

## Data Availability Statement

The datasets generated for this study are available on request to the corresponding author.

## Ethics Statement

The studies involving human participants were reviewed and approved by Research Ethics Committee of Helsinki University Central Hospital. The patients/participants provided their written informed consent to participate in this study.

## Author Contributions

SY and ES designed the research. SY and AN performed the research. SY, AN, and ES analyzed the data and modified the manuscript. SY wrote the original draft of the manuscript.

## Conflict of Interest

The authors declare that the research was conducted in the absence of any commercial or financial relationships that could be construed as a potential conflict of interest.
